# Evaluation of Cutting Forces and Roughness During Machining of Spherical Surfaces with Barrel Cutters

**DOI:** 10.3390/ma18153630

**Published:** 2025-08-01

**Authors:** Martin Reznicek, Cyril Horava, Martin Ovsik

**Affiliations:** Faculty of Technology, Tomas Bata University in Zlin, Vavreckova 5669, 760 01 Zlin, Czech Republic; mreznicek@utb.cz (M.R.); horacy00@seznam.cz (C.H.)

**Keywords:** milling, cutting force, barrel cutter, spherical surfaces, roughness

## Abstract

Barrel tools are increasingly used in high-precision machining of free-form surfaces. However, limited studies evaluate their performance specifically on spherical geometries, where tool–surface contact characteristics differ significantly. Understanding how tool geometry and process parameters influence surface quality and cutting forces in such cases remains underexplored. This study evaluates how barrel cutter radius and varying machining parameters affect cutting forces and surface roughness when milling internal and external spherical surfaces. Machining tests were conducted on structural steel 1.1191 using two barrel cutters with different curvature radii (85 mm and 250 mm) on a 5-axis CNC machine. Feed per tooth and radial depth of cut were systematically varied. Cutting forces were measured using a dynamometer, and surface roughness was assessed using the Rz parameter, which is more sensitive to peak deviations than Ra. Novelty lies in isolating spherical surface shapes (internal vs. external) under identical path trajectories and systematically correlating tool geometry to force and surface metrics. The larger curvature tool (250 mm) consistently generated up to twice the cutting force of the smaller radius tool under equivalent conditions. External surfaces showed higher Rz values than internal ones due to less favorable contact geometry. Radial depth of the cut had a linear influence on force magnitude, while feed rate had a limited effect except at higher depths. Smaller-radius barrel tools and internal geometries are preferable for minimizing cutting forces and achieving better surface quality when machining spherical components. The aim of this paper is to determine the actual force load and surface quality when using specific cutting conditions for internal and external spherical machined surfaces.

## 1. Introduction

Machining metallic and non-metallic materials is one of the widespread methods of transforming the original shape of a material—from a semi-finished product into a finished product. This transformation process—machining—adds value to the semi-finished product, which the manufacturer puts into it, thereby increasing it [1].

The greater this added value to the semi-finished product, the greater the price of the product itself. Furthermore, other aspects, such as machine tools and overhead costs, are entering into this evaluation process. All manufacturers aim to machine efficiently, minimizing machining costs. Several machining methods, which have different goals, then enter this production process—for example, removing material at the highest rate possible within the shortest timeframe during roughing operations [1].

The second major area of machining methods is finishing. Great emphasis is dedicated to the surface quality and the precision of the shape, according to the customer’s requirements. The simplest geometries for machining are planar, horizontal, and vertical surfaces, which are very easily machined by cylindrical tools. Inclined flat surfaces can also be easily machined with shaped tools or cylindrical tools using multi-axis machining. A large part of the tool is in contact with the workpiece in all these methods, and high surface quality and productivity are achieved. Due to the simple trajectory of the tool, the dimensional and shape deviation from the required differs only minimally and thus, meets the strictest requirements [1].

Ball milling is a contact machining process, the error of which is easily controlled due to the small area of the tool involved in cutting. Almost any surface can be machined like that because it is relatively easy to position the cutting tool, resulting in high accuracy [1]. However, it is very time-consuming. It may require multiple finishing passes when each pass only removes a small amount of material [2], making this technique relatively inefficient [1]. Another disadvantage is that the process produces a serrated finish, leaving a scallop between two consecutive passes [2].

Side milling with a barrel tool is a machining process with curved contact, which is more difficult to control due to the large area of the tool involved and the complex position between the tool and the machined surface. The side milling process can achieve much higher machining efficiency and a better surface quality than the face milling strategy [1]. Tool positioning is critical here, e.g., Yu et al. [3] focused in their article on a tool positioning method to achieve double contact in side milling with a barrel cutter, and Urbikan et al. [4] focused on the stability of contour maps for barrel cutters concerning tool orientation. Among other things, they discovered that for the simulations of angles of inclination and engages, the angle of inclination significantly affected dynamic cutting forces more than the angle of engagement.

The complications are free-form surfaces, spherical surfaces, or surfaces that are difficult to describe mathematically, such as the blades of aircraft engines and impellers. Generally known as freeform surfaces, the production of these surfaces was based on transferring the shape from a template. With the advent of CNC technology, these templates disappeared and were replaced by scallop machining. This machining method is based on the gradual machining of the material, often with a spherical tool, which gradually renders the product’s shape with a slight lateral displacement. However, this process is very time-consuming, and, with the increasing demand for surface quality and product precision, the time required for machining also increases disproportionately. Herraz and his team compared a ball and barrel cutter [5] and came up with a new method for deciding whether to use a ball or barrel cutter when machining free-form surfaces.

Part quality is characterized by two aspects: dimensional accuracy and surface roughness. While the first relates to macro-geometric errors and component interchangeability, surface roughness is related to micro-geometric errors and has essential implications for component lifespan and interoperability with other components [1]. Pelayo et al. [6] investigated the possibility of predicting surface roughness depending on the wearability.

During the milling process, the tool removes material in the form of chips and leaves traces on the surface of the workpiece. These subtle dips, peaks, and valleys create a roughness pattern on the workpiece that is specific to the type of operation used and is closely related to cutting parameters [6] such as cutting conditions, cutting geometry, workpiece specifications, and tool geometry choice. The basic parameters affecting the cut are the axial and radial depth of cut, feed rate, tool speed, and tool angle on the workpiece [7]. The cutting conditions then directly affect the state of the machined surface (surface texture, surface topography, surface residual stress, etc.) and subsequently affect the final properties of the workpiece [8].

Key mechanical properties imposed on components, such as stiffness, resistance to wear and corrosion, and fatigue strength, are affected by the roughness of the surface. It defines the surface’s ability to receive additional treatments and coatings. Thus, roughness affects functional properties such as sealing, wear, lubrication, contact resistance, noise, and vibration. Another important factor affecting surface quality is resistance to abrasion by the flowing medium. Therefore, controlling surface roughness is a crucial factor in the machining process [6].

Production of free-form surfaces on multi-axis CNC machines is a complex and expensive operation that produces many high-value parts (dies, pressing tools). For such operations, the choice of cutter type is crucial due to the significant impact of this choice on the quality of the obtained surface and the duration of the operation [5]. Meng et al. [9], in their article dedicated to the optimal selection of barrel cutters for the CNC machining of blades (BLISK), made a mathematical description of four types of barrel cutters for this purpose.

The disadvantage of machining with ball mills is the small contact area between the tool and the workpiece at the cutting point [1,6]. This disadvantage contributed to the development of more advanced tools with sophisticated cutting-edge profiles and geometries aimed at customized solutions [8].

Barrel cutters for machining shaped surfaces are not new inventions, but their full potential has been embraced recently with advances in machine control and CAM systems.

Machining with barrel-shaped tools, or tools with a significant edge curvature, is a very dynamically developing area. Artetxe and his team addressed this [10]; they constructed a model of mechanical cutting forces for barrel cutters. Their attention was focused on integrated blade rotors, the production of which is still very technologically demanding due to their complex geometry. Burek et al. [11] focused on testing the cutting forces of barrel cutters and concluded that the cutting forces have a large variability for different radii of curvature of the machined surfaces. According to them, this can lead to dimensional and shape errors. The values of the radii of curvature of the machined surface should thus be included in the design of the technological process. In their subsequent work [12], they came to the conclusion that it was necessary to change the width of the machining paths, which was reflected in changes in the cross-sectional area of the cut layer. Others who focused on cutting forces were Tamura et al. [13]; their work was devoted to the machining of conical surfaces.

This article aims to expand the field of machining with barrel tools by adding knowledge in the machining of spherical surfaces. This issue will be analyzed not only from the point of view of the shape (internal, external) but also from the dependence of the feed’s size and the radial depth of cut.

## 2. Materials and Methods

### 2.1. Material W. Nr. 1.1191

The used material was structural steel 1.1191 (W.-Nr.), DIN C45, or EURO C45E, suitable for refining and surface hardening. In the unhardened state, its tensile strength value (Rm) ranges from 530 to 620 MPa, and its yield strength value (Re) is 300 MPa. It is often used in the production of shafts of mining machines, pins, gears, connecting rods, piston rods, pins, pins, beds, connecting materials, latches, and rotors of screw compressors. Other properties are listed in Table 1, Table 2 and Table 3.

### 2.2. Machining

**Figure 1:**
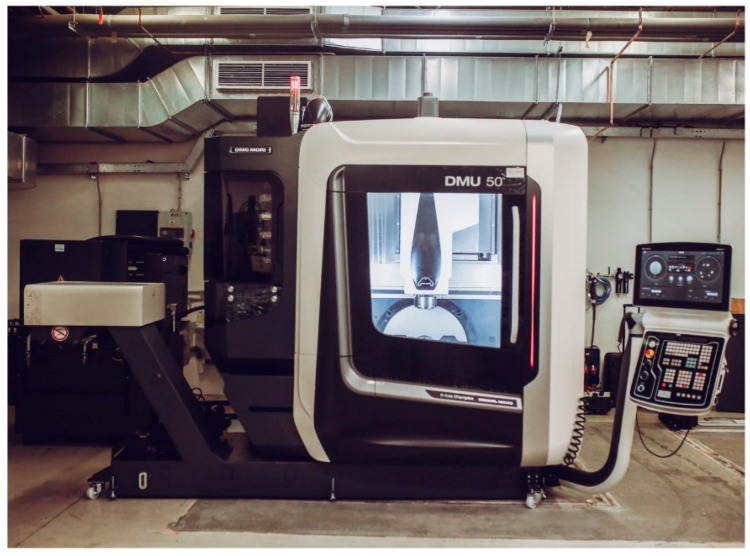
DMU 50.

A 5-axis machining center from DMG Mori (Figure 1) was used for experimental measurements. The rotations around the axis (B—35/+110°) (C—0–360°) enable continuous machining in five axes, representing ideal machining features when using barrel cutters. The highest (30 m/min) feed speed is sufficient for the chosen experiment. Maximum spindle speed (15,000 rpm) was limited for optimal cutting speed [15,16,17,18].

### 2.3. Tools

**Figure 2:**
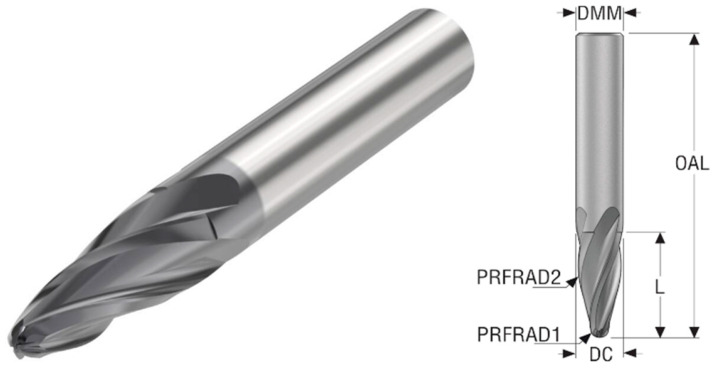
Barrel cutter R85 [19].

Two tools from the Seco manufacturer were selected for machining. For clarity, further in the article, the first that the manufacturer labels, JH734100X2R2R85.0Z4 SIRA, will be referred to as R85, and JH746100T2R2R250.0Z6 SIRA will be R250. Both tools fall into the barrel category but differ in the radius of curvature. The parameters of the R85 can be seen in Table 4, and its shape is shown in Figure 2.

**Figure 3:**
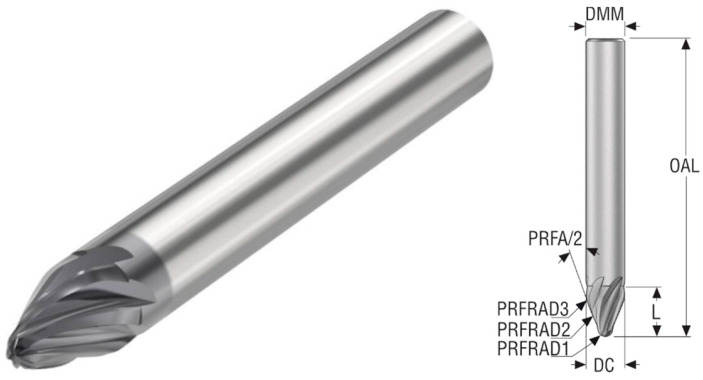
Barrel cutter R85 [20].

As mentioned, JH746100T2R2R250.0Z6 SIRA (R250) differs from the first mainly in the radius of curvature, the runout radius from the cutting radius PRFRAD3, and the defined angle of the division profile PRFA/2; its parameters are listed in Table 5 and is shown in Figure 3.

### 2.4. Milling Force Measurements

**Figure 4:**
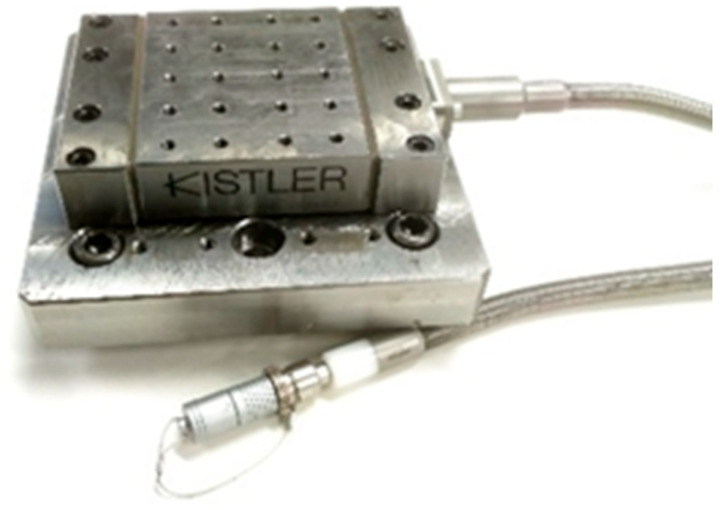
Dynamometer Kistler type 9129AA [21].

**Figure 5:**
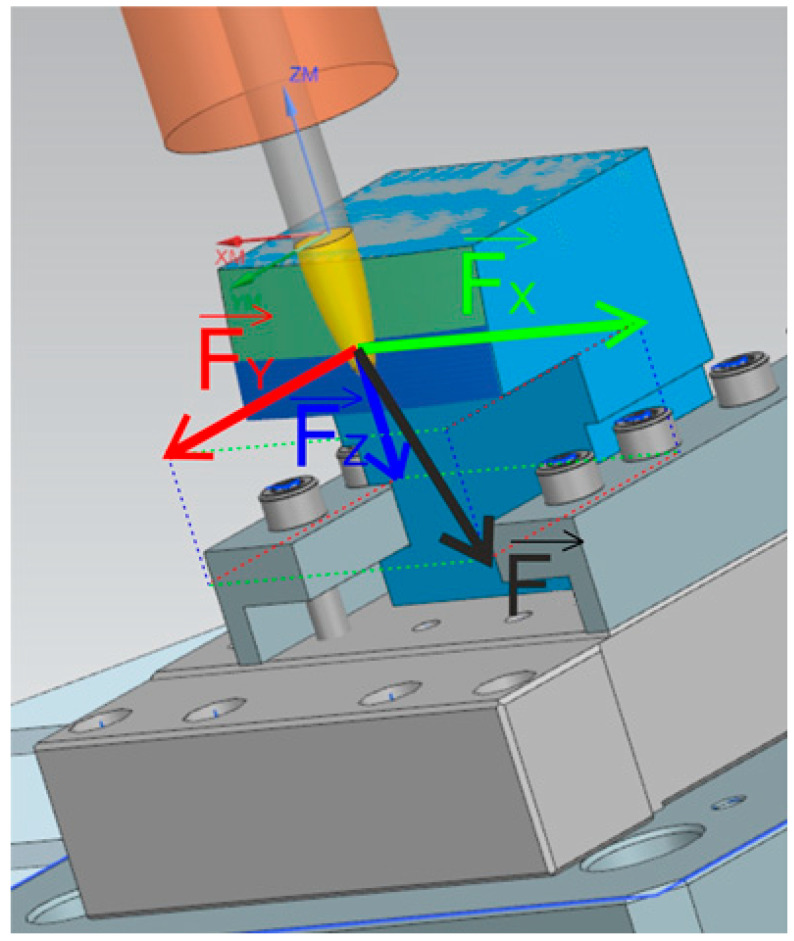
Test scene.

A Kistler dynamometer type 9129AA was used to record the cutting forces during machining. This dynamometer, with dimensions of 90 × 105 × 32 mm, allows for the work surface to be loaded up to 10,000 N for individual axes in both positive and negative directions. The connecting cable (Figure 4) transfers measured data with sensitivities of Fx ≈ 8.1 pc/N, Fy ≈ −4.1 pc/N, and Fz ≈ −8.1 pc/N (Figure 5) [21].

**Figure 6:**
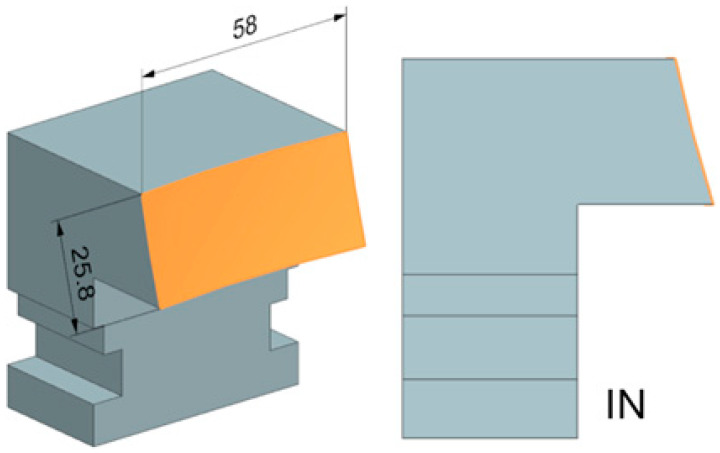

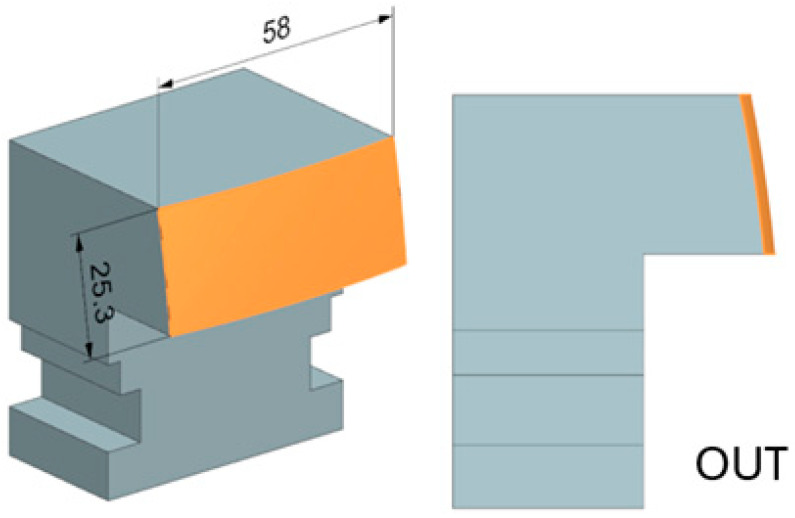
Test body IN and OUT.

Two simple spherical shape surfaces were designed and manufactured for the experiment. Both had a radius of curvature of 250 mm. The radius of curvature of the shaped surface is based on the radius of curvature of the tools used and its availability in the functional parts of the tool. Here, a larger radius was chosen, which can be machined even with a tool with a small rounding. The opposite curvature size would cause residual material that could not be machined. The resulting surfaces were machined from the inside (IN) and outside of the spherical surface (OUT). The final shapes of the test bodies can then be seen in Figure 6.

In order to make the force measurements as objective as possible, the original rough shapes of the test bodies were machined with standard tools using three-axis machining with finishing stock, like in practice. The roughing operations were always followed by milling with a tested tool without force recording to ensure the machined surface’s homogeneity while excluding the influence of the previous roughing operations on the finishing operations. This could manifest primarily in uneven material additions at the test face due to the so-called teeth after roughing.

After ensuring a constant allowance on the entire shape, the R85 and R250 test tools trajectories were designed and generated. The same operations were used to generate trajectories for both tools to exclude the influence of the trajectories.

CAM software NX version 1946 from Siemens was used as the path generation software. This program enables a full definition of a barrel cutter with all the necessary parameters. It also provides machining methods suitable for this type of tool. The most appropriate method was chosen from the machining operations offered: Variable Contour. With this operation, generating a trajectory with a multi-axis control position of the tool in relation to the workpiece is possible, thereby ensuring optimal contact with a surface.

**Figure 7:**
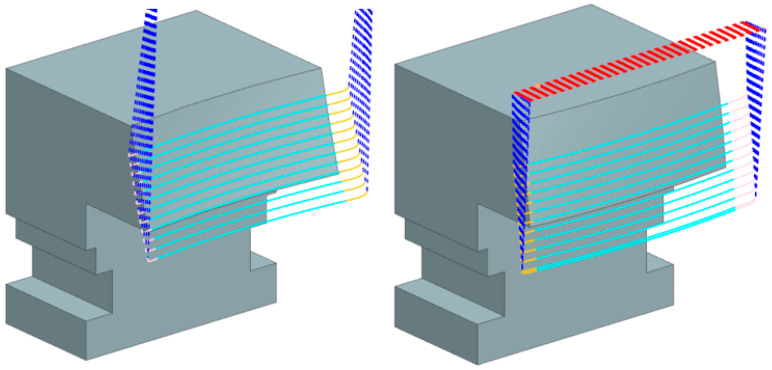
Trajectories for IN and OUT geometry.

Figure 7 shows the resulting trajectories. The path for the IN geometry is on the left, and on the right is a path for the OUT geometry. Both trajectories were optimized to maximize the similarity for both tools so that the trajectories do not introduce additional dependencies. As can be seen, the IN geometry required a higher engagement height so that the tilt angles were the same.

Changed variables were feed per tooth (f_z_) and radial depth of cut (a_e_). The values of both of these parameters were based on the manufacturer’s recommendations regarding the tool’s productivity and service life. The feed per tooth took values of 0.03 mm, 0.04 mm, and 0.05 mm, and the radial depth of cut also had three values: 0.2 mm, 0.4 mm, and 0.6 mm.

All experiments were performed at a constant cutting speed (230 m/min) with a cutting depth of approximately 2.5 mm.

## 3. Results

With the use of a Kistler 9129AA dynamometer, it was possible to record individual force components. Force component Fx acted in the direction of the tool feed, Fy in the direction of the radial feed of the tool (perpendicular to the feed rate), and Fz vertically to the clamping direction.

**Figure 8:**
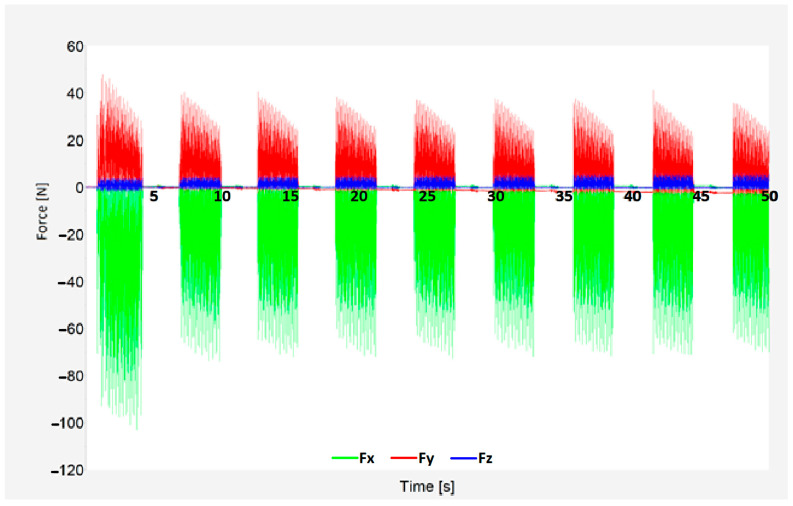
Cutting force results for *v*c = 230 m/min, *f*z = 0.03 mm, *a*_e_ = 0.2 mm, IN, R250.

An example of the results of the cutting forces can be seen in Figure 8. Force component Fx is shown in green, Fy in red, and Fz in blue. Scanning was carried out throughout the whole process to better and more accurately analyze machining. The first and last tool pass of the process was always excluded from the analysis because the tool was only partially in contact with the workpiece relative to the other passes, and constant cutting conditions could not be guaranteed. These passes showed differences in the display, and their inclusion in the results could affect the results. The rest of the passes showed excellent stability during the entire machining, proving that machining optimization was successful In Figure 8, due to only conventional milling being used (therefore pauses in the process), the graph shows the beginning and end of each tool pass as well as tool reposition using rapid feed to the beginning of the next engagement.

The forces’ most significant component was Fx, which took on values from 18 N to 150 N. In contrast, the Z-axis component ranged from 0 N to 11 N. For simplicity, only the total cutting force (Fc) will be presented. Its vector sum of all three components can be expressed using the formula below:

(1) $$\vec{Fc}=\sqrt{\vec{{Fx}^{2}}+\vec{{Fy}^{2}}+\vec{{Fz}^{2}}}$$

The following section presents selected results showing the influence of the radial depth of cut (ranging from 0.2 to 0.6 mm) and surface shape influence on total force (Fc).

**Figure 9:**
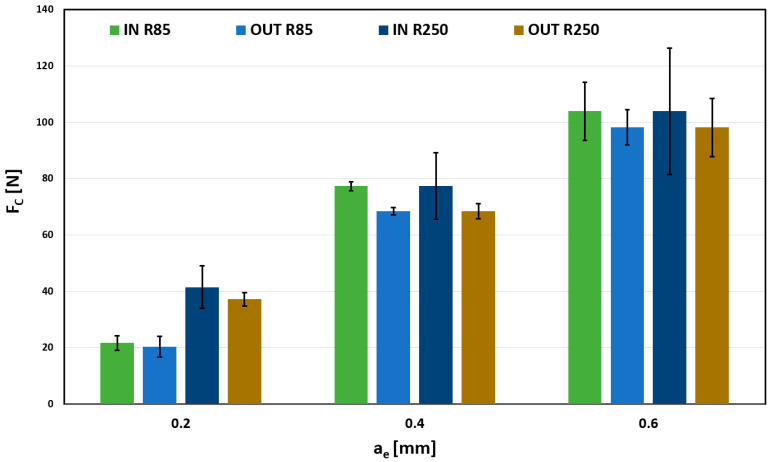
Influence of a_e_ on Fc for f_z_ = 0.03 mm/tooth.

Results for 0.03 mm/tooth feed are shown in Figure 9. The radial depth of cut’s influence on the total force is almost linear dependent. There are minor differences between the R85, R250, and the surfaces. However, these differences are within the measurement error, shown by the error bar, so it cannot be considered significant. Higher error segment values for force measurements during milling are common due to the chip formation process. Compared with other machining technologies, such as turning, the tool edge constantly starts and ends the cut, causing instability that increases with the depth of cut. By comparing individual values, taking into account the size of the error segments, it is not possible to clearly determine any dependence of the IN/OUT shape on the Fc parameter. The differences shown may be caused only by a random error that is smaller than the measurement error itself.

The influence of a surface on the total force Fc cannot be unequivocally confirmed because of plausible measurement deviations.

When comparing used tools, influence is more significant, especially in the case of a 0.2 mm depth, where total forces acting on the tool, respectively, on the workpiece, are almost twice as much for the R250.

**Figure 10:**
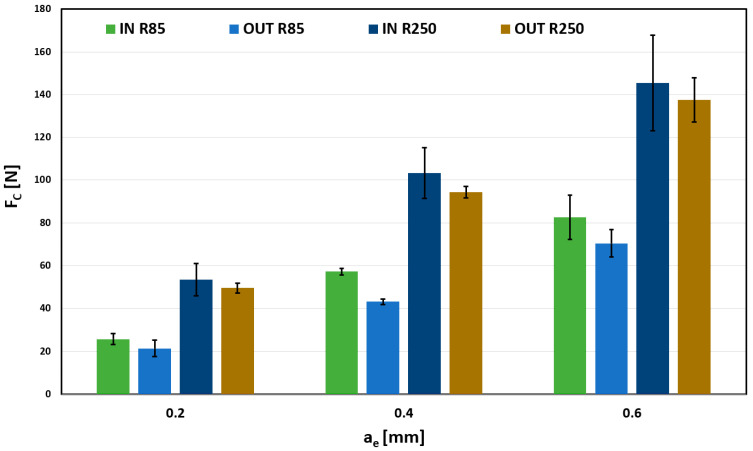
Influence of a_e_ on Fc, for f_z_ = 0.05 mm/tooth.

The same display as in Figure 9 was used to display the total cutting force Fc in Figure 10 for 0.05 mm/tooth feed. The results are almost identical to those in Figure 9; however, a more significant influence of the selected tool can be observed at 0.4 mm and 0.6 mm radial depth of cut. Changing the curvature of the tool from 85 mm to 250 mm causes an increase in the total cutting force to approximately double, which can significantly affect the tool and the load on the surface.

**Figure 11:**
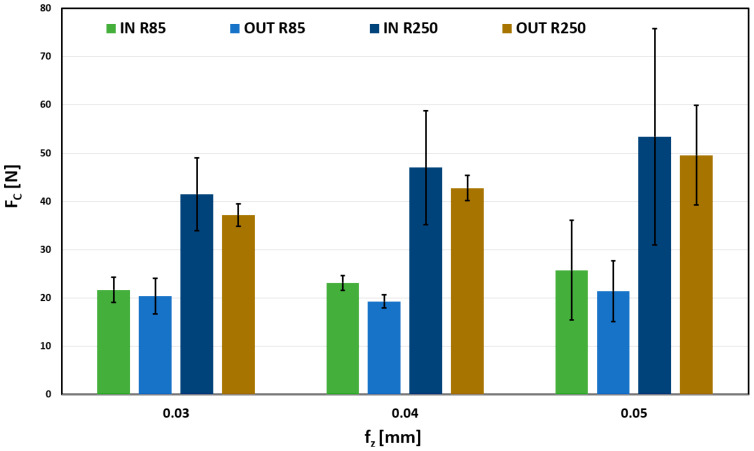
Influence of fz on Fc, for a_e_ 0.2 mm.

When comparing the effect of the feed per tooth (f_z_), for the values from 0.03 to 0.05 mm, shown in Figure 11, significant differences for the used tools can be seen. These differences were manifested in all observed depth values, always with approximately double the value.

The influence of the feed per tooth on the total forces is not significant; for all its values, the forces reach approximately 20 N. However, a significant force increase can be observed for a larger radius tool (R250 mm). Forces are doubled compared to a tool with a lower radius. This increase could negatively affect the quality of the product.

**Figure 12:**
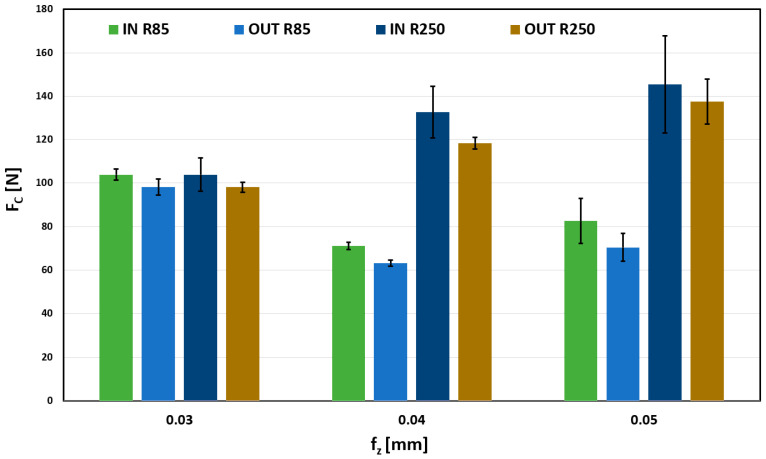
Influence on fz on Fc for a_e_ 0.6 mm.

Largest total forces were obtained when machining when a maximum radial depth of cut (0.6 mm) was used; the results of which are shown in Figure 12. Very balanced forces can be observed for the 0.03 mm/tooth feed. Thus, the choice of tool and surface shape are negligible. However, a more significant difference between other feeds (0.04 and 0.05) can be observed, mainly in dependence on the tool. A tool with a larger curvature radius causes a significantly greater force load than a tool with a smaller radius of curvature. Observed trends in previous measurements are thus confirmed.

During the experiments, the quality of the machined surfaces was also evaluated. The shape of the machined surface and the direction of tool paths determined the direction of roughness. It was in the direction of the tool axis, i.e., perpendicular to the tool paths. Edge areas of the evaluated surfaces were excluded from the roughness measurement. The length for evaluating roughness parameters was set to 4 mm, with the number of evaluated lengths being 5.

The tools and their pre-determination for machining shaped surfaces presuppose new approaches not only for the manufacturing of these surfaces but also for evaluating surface quality. For these reasons, the standard roughness parameter, Ra, was abandoned, and the evaluation focused on parameter Rz, which is mainly used in areas where higher demands on surface quality are placed; it is based on the component’s function or the surface evaluation. Advantages include a higher sensitivity to extreme peak heights compared to the Ra parameter while maintaining the possibility of averaging according to the DIN 4768 standard against the Rt parameter.

**Figure 13:**
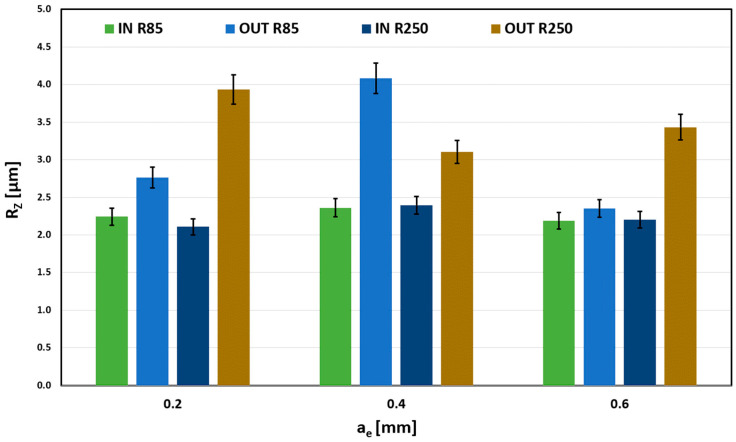
Influence of the a_e_ on Rz for f_z_ = 0.03 mm.

Figure 13 shows the evident influence of the machined surface shape (IN and OUT). The outer spherical surface always has higher values of the Rz parameter, which is caused by a smaller contact area (intersection of two spherical surfaces) that leaves higher scallops. In contrast, the inner spherical surface (IN) had a better contact surface due to the radius of curvature of the tool, and the transitions between individual cuts are smoothed while maintaining a constant depth of cut.

A mechanical model of machining with barrel tools can describe the contact surface issue even better, because it deals with the intersection of two curved bodies, which better represent the actual size of the chips being removed.

To determine the dependence and degree of correlation, a comparison of the measured data can be used with regard to the size of the error segments. Here, different error segment bands can be observed, whose mutual position proves the significant influence of the parameter a_e_ on the evaluated parameter Rz.

The Rz parameter’s dependence on the radial depth of cut (a_e_) size is negligible for the R85 tool and the inner spherical surface. Differences are noticeable for the outer curvature (OUT); the smaller contact area causes that.

**Figure 14:**
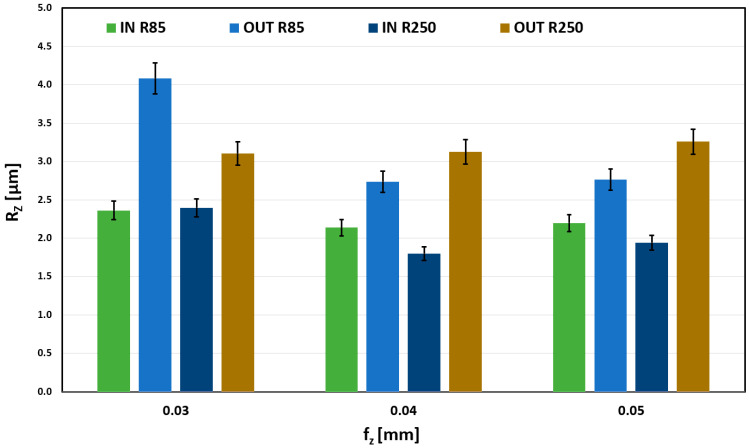
Influence of fz on Rz for a_e_ = 0.4 mm.

The effect of the feed per tooth on Rz is shown in Figure 14. Very stable results can be seen for the OUT geometry and R250 tool. Machining the outer surface with R85 proved to be stable for higher feeds (0.04 mm/z and 0.05 mm/z). For the lowest (0.03 mm/z), the surface quality worsens.

With the R85 tool and external shape machining, an abnormal increase in the RZ roughness parameter is observed for a feed rate of 0.03 mm/tooth. This increase was attributed to a combination of the convex machined shape and a tool with a small radius of curvature, resulting in a smaller contact area and a small feed rate, which could have caused tool vibration. This trend is also observed at feed rates of 0.04 mm/tooth and 0.05 mm/tooth, but it is not as pronounced as it is at the limit feed rate of 0.03 mm/tooth.

**Figure 15:**
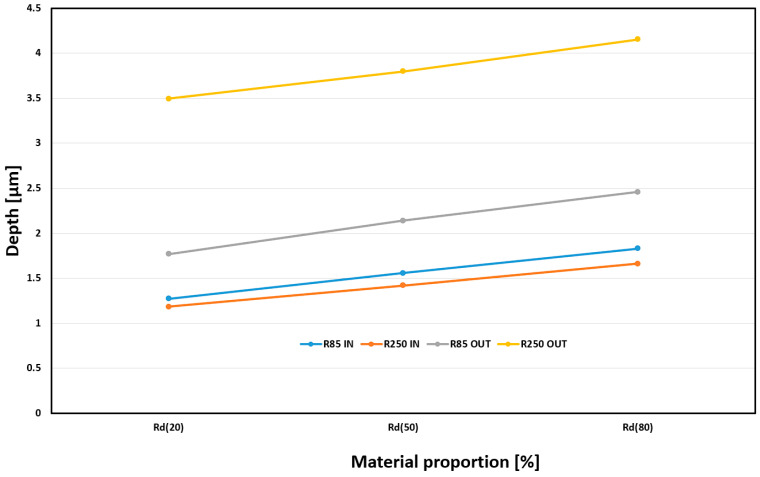
Influence of tool choice and geometry shape on Rd for a_e_ = 0.2 mm and f_z_ = 0.03 mm.

The final monitored surface quality assessment parameter was material proportion (Rd). Monitoring surface wear over time is desirable due to the expected application of the machined parts. The results of this observation can be seen in Figure 15. The monitored parameter was the depth of the required material portion. Based on practice, material shares were set for values of 20%, 50%, and 80%. These material content values can be defined in practice as gradual surface wear during the component’s life cycle. The 0–20% range is often referred to as the running-in period, when the highest peaks are leveled off in the first phase of use. The second phase, 20% to 80%, is associated with the actual use of the part and natural material loss, and the final phase, 80% to 100%, is associated with surface destruction and component failure. A significant influence of the machined shape on Rd can be observed. The greatest depths can be observed for the OUT shape machined by R250. On the other hand, the smallest depth of the proportion for all observed values is at both IN shapes. All combinations of machining (R85, R250, IN, OUT) have rising trends with similar growth and no effect on the type of geometry.

As can be seen from the measurements above and the principle of the tool’s use, these tools are outperforming the widely used ball tools in terms of their performance. Barrel tools enable more efficient machining in the form of larger spacing, thereby significantly reducing production times while maintaining surface quality requirements.

## 4. Conclusions

This article deals with the issue of machining with barrel tools, characterized by their large radius of curvature of the blade. Two tools with different radii of curvature, R250 mm and R85 mm, are compared here. Milling was performed under varying cutting conditions except for the cutting speed, which was set to 230 m/min.

W. Nr 1.1191 was chosen as the test material, which is among the most common materials for machining due to its mechanical properties and good machinability. The test specimens’ overall shape was optimized to exclude the effect of parasitic forces arising, for example, on the face of the tool or its shaft. These shaped surfaces can be part of thin turbine blades, which are susceptible to deformation during machining, negatively affecting their production accuracy.

When evaluating the results, there was a non-negligible influence of the tool choice, or rather its radius, on the cutting forces. This effect turned out to be less significant at the smallest feed per tooth used (0.03 mm). In other cases, the size of the total cutting force increased to double, highlighting the importance of choosing the right type of tool. Contrarily, the influence of the inner or outer shape proved to be less significant, where the change in the size of the contact area of the tool due to the shape is practically negligible, and in practice, it is not necessary to take it into account. Change in the radial depth of cut (a_e_) size significantly affects the size of the total cutting forces. This also corresponds with the general conclusions and recommendations for standard cylindrical tools.

Based on the measurements, it can be recommended to choose a tool with a smaller radius of curvature, a smaller radial depth of cut, and higher feed per tooth to minimize the total size of the cutting forces. The measured data can also be applied in practice and used to predict surface quality under the same selected machining conditions. Surface shape (internal or external) has a small part in the force’s magnitude. So, it is unnecessary to consider it when designing these surfaces. As for surface quality, internally shaped surfaces should be preferred over external ones with regard to the curvature radius of the tool.

## Figures and Tables

**Table 1:** Chemical composition of W. Nr. 1.1191 [14].

Element	Proportion (%)
C	0.42–0.50
Si	max 0.40
Mn	0.50–0.80
P	max 0.03
S	max 0.035
Cr	max 0.40
Ni	max 0.40
Mo	max 0.10
Cu	max 0.30

**Table 2:** Physical properties of W. Nr. 1.1191 (20 °C).

Property	Unit	Value
Density	(g/cm^3^)	7.9
Specific heat capacity	(J/kg K)	500
Thermal conductivity	(W/mK)	15
Electrical resistivity	(Ω mm^2^/m)	0.73

**Table 3:** Mechanical properties of W. Nr. 1.1191 (20 °C) [14].

Property	Unit	Value
Hardness HB 30	(HB)	≤255
0.2% Yield strength Rp	(N/mm^2^)	≥300
Tensile strength Rm	(N/mm)	530–620
Elongation A_5_	(%)	≥18

**Table 4:** Parameters of the JH734100X2R2R85.0Z4 SIRA [19].

Property	Unit	Value
Cutting diameter (DC)	mm	10
Shank diameter (DMM)	mm	10
Profile radius 2 (PRFRAD2)	mm	85
Profile radius 1 (PRFRAD1)	mm	2
Face cutting edge count		4
Overall length	mm	72
Flute Helix Angle	deg	20
Depth of cut maximum in feed direction side	mm	22.3
Cutting geometry type		JH734

**Table 5:** Parameters of the JH746100T2R2R250.0Z6 SIRA [20].

Properties	Unit	Value
Cutting diameter (DC)	mm	10
Shank diameter (DMM)	mm	10
Profile radius 3 (PRFRAD3)	mm	5
Profile radius 2 (PRFRAD2)	mm	250
Profile radius 1 (PRFRAD1)	mm	2
Face cutting edge count		6
Overall length (OAL)	mm	75
Flute Helix Angle	deg	20
Depth of cut maximum in feed direction side	mm	9
Cutting geometry type		JH746
Profile angle divided by 2 (PRFA/2)	deg	20

## Data Availability

The original contributions presented in this study are included in the article. Further inquiries can be directed at the corresponding author(s).
